# Recapturing the History of Surgical Practice Through Simulation-based Re-enactment

**DOI:** 10.1017/mdh.2013.75

**Published:** 2014-01

**Authors:** Roger Kneebone, Abigail Woods

**Affiliations:** 1 Department of Surgery and Cancer, Imperial College London, Clinical Skills Centre, 2nd Floor Paterson Building, St Mary’s Hospital, Praed Street, London W2 1NY, UK; 2 Department of History, Room C8B, East Wing, Strand Building, King’s College London, London WC2R 2LS, UK

**Keywords:** Surgery, Expertise, Tacit Knowledge, Simulation, Re-enactment, Twentieth Century

## Abstract

This paper introduces *simulation-based re-enactment* (SBR) as a novel method of documenting and studying the recent history of surgical practice. SBR aims to capture ways of surgical working that remain within living memory but have been superseded due to technical advances and changes in working patterns. Inspired by broader efforts in historical re-enactment and the use of simulation within surgical education, SBR seeks to overcome some of the weaknesses associated with text-based, surgeon-centred approaches to the history of surgery. The paper describes how we applied SBR to a previously common operation that is now rarely performed due to the introduction of keyhole surgery: open cholecystectomy or removal of the gall bladder. Key aspects of a 1980s operating theatre were recreated, and retired surgical teams (comprising surgeon, anaesthetist and theatre nurse) invited to re-enact, and educate surgical trainees in this procedure. Video recording, supplemented by pre- and post-re-enactment interviews, enabled the teams’ conduct of this operation to be placed on the historical record. These recordings were then used to derive insights into the social and technical nature of surgical expertise, its distribution throughout the surgical team, and the members’ tacit and frequently sub-conscious ways of working. While acknowledging some of the limitations of SBR, we argue that its utility to historians – as well as surgeons – merits its more extensive application.

## Introduction

This paper introduces simulation-based re-enactment (SBR) as a method of recreating, recording and investigating the recent history of surgical practice. Working in collaboration, a surgeon and a historian brought together retired surgical teams (comprising surgeon, theatre nurse and anaesthetist) within a simulated operating environment which mirrored the essentials of a clinical setting without reproducing every detail. The teams performed operative procedures that are no longer used routinely but remain within living memory. They also instructed present-day surgical trainees in operative techniques. Captured by video recording, and supplemented by multiple interviews, their activities were placed on the historical record and subjected to critical historical analysis.

The importance of this study is primarily methodological. Deriving from the collaborative work of a surgeon and a historian,^1^ it offers a new approach to documenting the history of surgical practice, and reveals the value of these documents for illuminating the nature of surgical expertise. Hitherto, historians of surgery have relied primarily on textual sources. Consequently, the people and problems that generated the largest quantity of texts have attracted the most historical attention, resulting in a profusion of histories of antiseptic surgery, anaesthesia, war-time surgery and surgeons’ biographies.^2^ While valuable, these histories offer few insights into what actually happened within the closed environment of the operating theatre. As Andy Warwick noted in 2005, little is known about how surgical procedures were devised, performed, improved and taught, and how these activities shaped, and were shaped by support staff, patients, instruments, machines and surgical skills.^3^

This omission is partly addressed in histories written by surgeons, who offer an ‘insider perspective’ on events within the operating theatre.^4^ However, such accounts necessarily privilege the interests and perspectives of their authors. Although by the mid-twentieth century surgery had changed from an individual pursuit into a form of teamwork,^5^ surgeons’ insights remain focused on their own roles, and neglect the other members of the team. Surgeons also assume a large amount of contextual knowledge on the part of the reader. When writing on instrumentation and operative technique they do not explain matters such as the choice and use of instruments, handling of tissues, collaborative actions with other team members, or the characteristics of a good surgeon. Moreover, they rarely comment explicitly on how surgical practice was shaped by external influences such as institutional and government policies, economic pressures, managerial regimes and patient demands.^6^

Accounts that utilise surgeons’ testimony while locating it within historical context have started to address these deficiencies.^7^ However, difficulties remain because throughout history, much surgical practice has not been subjected to verbal description. This is also a problem for the history of science, technology and medicine more broadly. In the contemporary context, it is possible to use ethnography to illuminate the unspoken social and practical dimensions of these fields, the embodiment of knowledge in instruments, and its derivation from collective rather than individual efforts.^8^ However, some controversy has surrounded the application of ethnography to surgery. Authors have disputed the validity of findings, and whether ethnographers should aim to understand the surgeon’s point of view or to provide an outsider perspective.^9^  Nevertheless, their work reveals that the expert performance of surgery involves a complex amalgam of technical skill (requiring high levels of dexterity, precision and fine motor coordination), communication, situational awareness, the ability to respond effectively to rapidly changing conditions and a range of other attributes.

Despite popular stereotypes of the lone ‘heroic surgeon’, ethnographers have shown that surgical expertise is distributed across the historically neglected surgical team, whose performance is much more than the sum of its parts. Its members coordinate the resources of the operating theatre in time and space, thereby enabling the surgeon to assume and power and control. Expertise is expressed in their collaborative ways of working, which rely on complex unspoken communications, relationships, and interactions. Members also draw upon a huge repertoire of automated, tacit and shared ‘ways of doing’ that extend to aseptic rituals, technical procedures, appropriate behaviours and the use of space.^10^

It is by participating in these teams that present-day trainee surgeons move from being peripheral participants to central players. They learn, by osmosis, the tacit knowledge, embodied practices, self-discipline, gestural language and codes of conduct required to perform surgical operations. Training is concerned as much with developing the social skills and professional values of the surgeon as with learning the necessary visual and motor skills.^11^  Indeed, Bosk’s analysis of surgical training in the 1970s noted that trainees’ technical and judgemental errors were often tolerated more than their failure to understand the norms of the group, or the senior surgeon’s codes of conduct.^12^

Some of the expertise acquired in training may be impossible to verbalise. It thereby conforms to the definition of tacit knowledge advanced by Collins. For Cambrosio and Keating, however, tacit knowledge includes that which could be articulated but which in everyday contexts is left unsaid, perhaps because it is seen as trivial or already widely known. As Schlich shows in his account of the AO system of fracture care, training is an important context for the verbalisation of such knowledge.^13^ The ethnography of training therefore offers especially important insights into aspects of surgical expertise that normally remain tacit.

It is rather more difficult to capture the tacit aspects of past surgical practices. Their non-verbal and frequently sub-conscious aspects are not made explicit in primary historical texts, and cannot be uncovered and captured by individual and group oral histories.^14^ Some insights into the technical aspects of expertise are offered by surviving training videos, and the instructions and illustrations of surgical text-books, especially if one follows Hirschauer in regarding the surgical process as an attempt to create the textbook body by turning the patient into a passive object, and identifying, isolating and making visible the relevant parts.^15^ However, in focusing upon the surgeon’s technical actions within the operative site, these source materials exclude the broader social environment of the operating theatre, and the ways in which the surgeon’s expertise intersected with, and was supported by that of the team.^16^ Their roles could potentially be illuminated by the history of surgical instruments, for as Ghislaine Lawrence argued in 1992, ‘surgical instrument design has certainly been affected by the presence or absence of assistants during operations’.^17^ However, while other medical technologies have attracted historical attention,^18^ her call for ground-up studies of the everyday practices of instrument users has not been answered.

In proposing simulation as one way of capturing the historical elements of surgical expertise, we draw inspiration from two existing methodologies: historical re-enactment, and the use of simulation within present-day surgical education. We will start by discussing these methods and how they informed our approach to surgical history. We then describe the use of SBR to recreate, and capture for the historical record, a particular surgical operation: cholecystectomy in the 1980s. Next, we use this evidence to make observations on the nature, application and acquisition of surgical expertise. In conclusion, we reflect on the benefits and drawbacks of SBR as a means of reconstructing and recording the past, and how our findings might prove valuable to surgeons as well as historians.

## Re-enactment and Simulation

The re-enactment of historical events is an increasingly popular activity. Usually applied to events such as battles or social practices which no longer fall within living memory, it offers a means of engaging the public with their pasts, thereby advancing the agendas of public history.^19^ Advocates of this form of re-enactment often find it difficult to convince their peers that it is more than ‘merely the present in funny dress’. Cook points out that as a narrative method, and an investigative tool directed towards learning about (rather than simply dramatizing) the past, re-enactment also encounters problems of *analogy* (the difficulties of mapping subjective experience of present-day participants onto a historical situation), of *focus* (which is necessarily selective), and of privileging the *emotional engagement* of participants and audiences over analytical objectivity.^20^  Nevertheless, he argues that it offers an important route to understanding the past, because ‘re-enactments force participants and audiences to consider the material, environmental and cultural constraints under which all lives are lived.’^21^

It is these ‘material, environmental and cultural constraints’ that we have tried to address with SBR, by triangulating and cross-checking our provisional re-creation of past operative settings with the collaborative participation of those who were there at the time. This approach distinguishes SBR from re-enactments based on the more distant past, and confines its scope to operations that remain within the experience of people alive today.

We also draw on historians’ and philosophers’ attempts to re-enact historical experiments. Their work ranges from the replication of alchemical experiments^22^ to the history of physics,^23^ the seventeenth-century work of Malpighi,^24^ the investigations of James Joule,^25^ and eighteenth- and nineteenth-century attempts to measure the boiling point of water.^26^ Relying, to varying degrees, on historical apparatus, contextual knowledge and archival material, scientific re-enactment has been pursued with a range of goals in mind: to know how experimenters reached their conclusions; to understand what they knew and how they thought; to recapture and test the veracity of forgotten findings for the purpose of informing present-day science; and – perhaps most importantly for the present study – to recapture the tacit dimensions of experimental practices. Although such approaches have not been applied specifically to procedures within the history of clinical medicine, they suggest ways in which simulation may help to recapture working practices and the embodied nature of expertise.

Several advocates of scientific re-enactment also support its use within present-day science education. They argue that as a form of ‘learning by doing’, participation in past experiments will not only educate students in the content of science, but also in its practices, processes and contexts.^27^ Although focused on present rather than past procedures, the use of simulation within clinical medical education has very similar goals. At its simplest, simulation employs physical or computer-based models to enable students to practise clinical procedural skills, such as suturing, blood sampling and placing urinary catheters, without endangering patients. It is not confined to individual procedures, but can also address complex team working.^28^ For example, anaesthetists have pioneered emergency management and team training by developing group activities around sophisticated computerised mannequins whose physiological responses can mimic important or rarely-encountered clinical situations and emergencies.^29^

Many universities and teaching hospitals now have advanced simulation centres, where full scale replicas of operating theatres and intensive care units allow teams to practise at regular intervals. Video-recording technology, debriefing facilities and highly developed educational programmes have made such centres pivotal elements of anaesthesia training. Until recently, however, it has been difficult to create surgical simulations which generate similar levels of engagement. This is mainly because of the challenge of recreating human organs that look and feel authentic. Kneebone has pioneered a number of innovative approaches to address this issue, thereby enabling training in operative procedures to be embedded within the wider socio-technical complexity of the operating theatre.^30^  By working with prosthetics experts from film and television he has developed surgical models made from silicon and other materials, creating highly realistic organs which secure high levels of engagement from participants. Simulated organs feel and handle like real human tissue, bleeding when appropriate and allowing a range of procedures to be performed. Kneebone has also pioneered combinations of cadaveric animal organs (for example, a pig’s liver and gallbladder) placed within or alongside a silicon human model.

A key advantage of such simulations is that they recreate the social, moving beyond the technicalities of operative surgery to encompass team working and collaborative behaviour. However, the scarce and costly nature of dedicated surgical simulation centres has confined their use mainly to the training of clinical teams. To address this issue, Kneebone has worked with clinicians and industrial designers to create low-cost, portable simulation environments (distributed simulation) that recreate key elements of a surgical setting without requiring the full panoply of a dedicated simulation centre.^31^ Selected contextual triggers (such as a small tripod-mounted operating lamp, a simplified representation of an anaesthetic machine printed as a conference banner and a background of recorded sounds) are sufficient to evoke a powerful sense of place. Lightweight wireless video cameras allow multiple views to be captured, providing close-up footage of operative technique alongside capture of team communication and interaction.

When coupled with realistic prosthetics and used by an appropriately garbed surgical team, these environments provide a highly compelling sense of being part of an operation. They also enable distributed simulation to be used in non-clinical settings, thereby opening up the closed world of the operating theatre to individuals who are not normally present. One important application has been in public engagement activities which enable lay audiences to experience and interact with the work of surgical teams.^32^ A second, highly novel application is described in this paper: the adaptation of distributed simulation for the investigation of past surgical expertise.

## Development of SBR as a Historical Method

We decided to apply SBR to one particular operation – cholecystectomy (removal of the gallbladder, usually for gallstones) – and selected 1983 as our index year. While the features of the operation were not specific to that year, they were broadly representative of an important era of post-war surgery which was then on the cusp of change. The specific date was chosen because it coincided with the creation of a full size replica operating theatre within the London Science Museum’s *Lower Wellcome Gallery*, where we sited some of our SBR.

Cholecystectomy was the fourth most common general surgical operation at that time, with over 36,000 cases per year being reported in 1978 in the UK. In its straightforward form as an elective procedure, the operation would be performed unsupervised by relatively junior trainees. Usually uncomplicated to perform, cholecystectomy constituted staple fare for surgeons at all levels of training, though at times it could tax the skills of the most experienced operator. The operation required a large incision under the patient’s ribs on the right side, allowing the anatomy to be displayed and the gallbladder removed. The wound was then closed and the patient would spend many days in hospital recovering before being discharged for further weeks of convalescence before returning to work.

This technique had changed little since the early twentieth century. Nine successive editions of Maingot’s *Abdominal Surgery,* published between 1940 and 1989, revealed fairly constant descriptions,^33^ which were echoed in a 1983 video recording of the operation prepared by surgeon Professor Harold Ellis for medical students.^34^ As noted above, these sources are primarily concerned with the technical details of the operation. The video camera is fixed on the operative site, while in Maingot’s books, key anatomical structures and instruments are shown in black and white illustrations produced by a medical artist. The surgeon’s gloved hand or fingers are sometimes shown, but no information is provided about the team, the social practices of the operation or the wider context within which it was carried out. The following passage, from Maingot’s chapter on cholecystectomy (7th edn, 1980) is typical:

After the [cystic] artery is divided the fatty envelope around the cystic duct is dissected clear, and the duct is traced to its junction with the common hepatic duct and the common bile duct. When there three ducts have been freed and displayed, an aneurysm needle threaded with a strand of 0 (m.4) chromic catgut is passed underneath the cystic duct, and this duct is ligatured almost flush with the main ducts.

By the late 1980s, a new technique of laparoscopic cholecystectomy (keyhole surgery) was being introduced. The gallbladder was removed through several small punctures in the abdominal wall, avoiding the need for a major incision. Hospital stays were dramatically shortened and there was much less post-operative pain. Keyhole surgery became widely adopted, and within a relatively short time the traditional approach of open surgery had been superseded except when complications arose. Today, almost all routine cholecystectomies in the developed world are carried out laparoscopically.^35^

**Figure 1:**
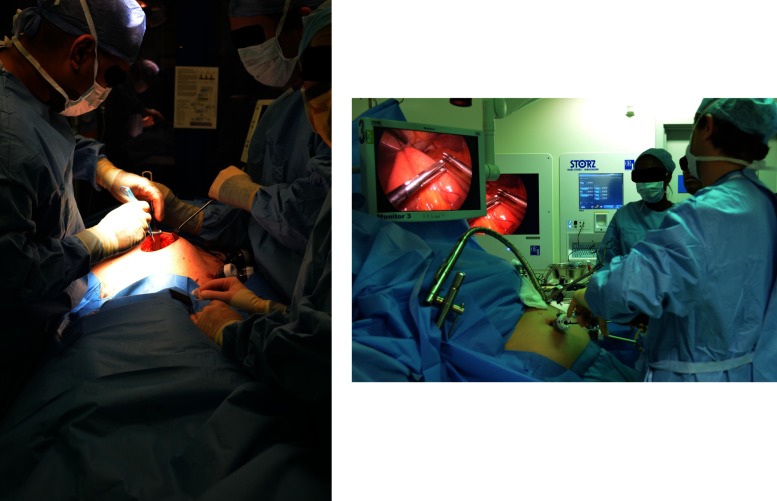
Open and laparoscopic surgery, 2013.

Our selected year, 1983, therefore represented the end of a long period of technical stability in this very common operation. It was also a period of stability in ward care and surgical working patterns. During the mid to late-twentieth century, each consultant surgeon had a ‘firm’ of more junior clinicians, which trainees would join at intervals. They worked with ‘their’ anaesthetist and ‘their’ theatre sister for years and sometime decades. In 2003, the introduction of the European Working Time Directive dramatically reduced the long working hours of junior clinical staff. A key consequence was a change from the ‘firm’ structure to shift work, leading to a dissolution of previously stable social structures and educational groupings.^36^ At the same time, profound changes in the structures of clinical training and in the relationship between publics and the professions radically altered the status quo.^37^

This combination of changes in the techniques and social structures of surgery means that the expertise and ways of working that characterised the past performance of operations like open cholecystectomy are now in danger of being lost. As highlighted above, these practices involved many tacit and subconscious dimensions. Once they pass beyond lived experience, they will prove difficult if not impossible to reconstruct, thereby putting these very important aspects of the history of surgery beyond the reach of the historian. However there are still teams alive today – many having qualified during or soon after the Second World War – who performed this operation throughout their professional careers. Using SBR we sought to place their collective expertise on the historical record and to analyse its nature and acquisition.

Some of our SBR was conducted within the London Science Museum’s *Lower Wellcome Gallery*. This contains a replica operating theatre that was closely modelled on a St George’s Hospital surgical suite and created with input from a leading surgeon and theatre sister of the time.^38^ It was intended, in 1983, to illustrate the state of the art of contemporary surgery, in contrast to the historical practices displayed elsewhere in the gallery. Although designed as a cardiac surgery operating theatre, it contained much material relevant to our SBR, including authentic surgical instruments, operating lamp, operating table and anaesthetic machine. Working closely with the Museum’s curators and conservators, we reconfigured this space and its contents to resemble how a general surgery operating theatre looked at the time.^39^ To assist this process, we took one of our surgical teams (see below) to view the Science Museum’s extensive reserve collection of objects at Blythe House, London.

To see Professor Stanley Feldman demonstrating an anaesthetic machine, view supplementary movie 1 (available at http://dx.doi.org/10.1017/mdh.2013.75).

Members’ encounters with museum artefacts were video-recorded. They provided strong support to our preliminary hypothesis that physical objects such as surgical instruments, anaesthetic equipment and surgical garb might act as triggers for recollection, activating embodied memories which had temporarily passed beyond conscious recall, and would therefore remain inaccessible to interview and conversation. This was especially apparent with several team members present, which allowed conversations to ensue. The authenticity of the operating environment was further enhanced by taping and replaying operating theatre sounds (including the regular rise and fall of the ventilator bellows). A human form (mannequin) was placed on the operating table, and the operative site simulated through the use of a hybrid model featuring a cadaveric pig liver and gallbladder within a silicon abdominal cavity with surrounding organs.

Recognising that it would not be possible to use the Science Museum’s operating theatre on a regular basis, and that accessing full-immersion simulation facilities within existing surgical education centres would be difficult and expensive, we constructed an additional site for SBR within Kneebone’s research group at Imperial College London (St Mary’s Campus). This drew on Kneebone’s research into distributed simulation as a means of securing high levels of perceived realism at minimal cost through ‘selective abstraction’ of key aspects of a surgical setting (see above). Selected artefacts were positioned within an inflatable enclosure, coloured to represent an operating theatre. Surgical and anaesthetic equipment from the 1980s was placed in position and care was taken to recreate as rich a sensory environment as possible (including sound). Lightweight wireless video cameras were placed to capture multiple viewpoints.

One re-enactment session was performed in each location, each involving a different surgical team. The teams were recruited through personal contacts with clinicians who had retired at or before 1989, the Royal College of Surgeons’ volunteer network, and their erstwhile colleagues and team members. Each contained a surgeon, an anaesthetist and a theatre nurse who had worked together for decades. The first team was Professor Harold Ellis (surgeon), Professor Stanley Feldman (anaesthetist) and Sister Mary Nieland (theatre sister) from the Westminster Hospital, London, a major teaching hospital. Ellis was Professor of Surgery until his retirement from clinical practice, and is renowned nationally and internationally as a teacher, surgeon, anatomist and historian. Feldman is an eminent clinician and clinical researcher, who made major contributions to many areas of anaesthesia over a long and distinguished career. Nieland is an experienced theatre sister who held senior hospital positions. The second team was Mr John Black (surgeon), Dr Bruce Roscoe (anaesthetist) and Sister Julia Radley (theatre sister) from Worcester Hospital, a District General Hospital. Black was the President of the Royal College of Surgeons of England until 2011. He worked closely with Roscoe and Radley over many decades.

Additional team members were composed of surgeons in training from Kneebone’s research group, together with medical students at Imperial College. By simulating historical training patterns, their presence encouraged retired surgeons to verbalise practices that would otherwise remain tacit. In-depth individual and group interviews with team members (audio-recorded and annotated in line with British Library Oral History guidelines) were conducted by Kneebone, providing a baseline record of participants’ recollections of surgery from the time in question. These interviews captured a general sense of key stages in the operation, but little detail relating to the social practices of surgery.

Each team was asked to complete an operating ‘list’ including two cholecystectomies, the first performed by the senior surgeon, the second performed by a present-day surgical trainee under the direct supervision of the senior surgeon. In each case the operating surgeon was assisted by one or two ‘juniors’; the anaesthetist (accompanied by present day students or anaesthetic trainee) standing at the patient’s head; and the theatre sister (scrub nurse) standing at her trolley next to the surgeon. Sessions were extensively documented with still photography and video recording. We commissioned a professional recording crew (Consortium TV) to capture multiple views of the operations as they were taking place. These included a long view of the operating team, roving close-ups captured by a hand-held camera operator, and details of the operative field via a static camera positioned close to the operating lamp. Multiple audio tracks were recorded, allowing dialogue within subgroups of the surgical team (e.g. surgeon/assistant; surgeon/nurse; and anaesthetist/assistant) to be captured. A full record of all recordings has been created and stored securely. Raw footage was edited and collated for initial analysis, creating relatively brief clips of each phase of the operation which could be used during individual and group review sessions with the participants.

To see Professor Harold Ellis’s team operating, view supplementary movie 2 (available at http://dx.doi.org/10.1017/mdh.2013.75).

In this way, we sought to capture the general behaviours of team members; the manifestations of surgical technical expertise; the influence of personalities, hierarchical dispositions and social interactions on ways of working; and how these matters were communicated through surgical training. Each re-enactment was followed by a period of group reflection, intended to test the authenticity of the simulation, and to trigger cumulative, collective recollections of past working practices.

## Findings: The Nature of Surgical Expertise

As already noted, while ethnographic and educational studies have highlighted the present-day dimensions of team-based surgical expertise, they reveal little about how the social and technical nature of this expertise has changed over time. By analysing the video recordings of our surgical re-enactments, in conjunction with pre- and post-re-enactment interviews, we were able to draw some preliminary observations on the historical nature and use of expertise. These observations incorporate aspects of working relationships between members of the surgical team, their use of space, the integration of roles and responsibilities, and the technical aspects of performing a particular operation.

One striking feature of these re-enactments was the ease with which members of the surgical team settled into their accustomed roles. Although it was many years since they had last worked together, and the operating environment was different in certain respects (most notably in the lack of human patient), they quickly dressed in their theatre garb (gowns and gloves) and assumed their customary positions around the operating table. A relaxed atmosphere and light-hearted banter (often referring to shared experiences from the past) showed a strong sense of team awareness. To us as observers, a striking characteristic was the ability of the team to integrate multiple tasks with no apparent effort, and to incorporate other team members (such as trainees and students) into both practice and teaching. These impressions were confirmed in post-re-enactment interviews, when participants commented that although the setting was not completely authentic, it allowed them to work with their colleagues very much as they had on previous occasions. This suggests that surgical and educational practices were deeply ‘ingrained’ in all members of the surgical team, and rapidly resurfaced in response to appropriate external stimuli.

The theatre sister (scrub nurse) played a crucial role in assisting the work of the surgeon, thereby demonstrating the distributed nature of surgical expertise. Standing at his side, she arranged her tray of instruments neatly at the outset, positioning them in accordance with their likely usage, and ensuring that handles were aligned. She adopted a characteristic posture, keeping her attention fixed on the operative field, while pulling her elbows close to her body to avoid intruding on the surgeon’s space. She responded quickly to his requests for particular instruments or simply knew from his gestures what was required. When the surgeon omitted to articulate the name of an instrument, saying ‘Sister, give me a 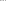 [tailing off]’, she either prompted him by saying the name herself, handing him the relevant item, or suggested various instruments that might suit his needs. At other times, she would glance at the operative field and pick up an instrument which had not yet been asked for, holding it in readiness for up to two minutes. Sometimes she had several such instruments to hand, able to move instantly to the one required. As the operation proceeded, she deftly pulled the instruments and swabs that he had discarded away from the operative field. Instruments that would be needed again she repositioned so that the handles were ready for him to grasp.

The anaesthetist was also a crucial part of the dynamic. Physically, he was somewhat removed from the surgeon and nurse, standing by the head of the patient and taking frequent measurements of pulse and respiration. In both teams he exchanged banter with the surgeon (though seldom the theatre sister (scrub nurse)), reflecting their longstanding professional (and sometimes social) relationship. He re-assumed his customary responsibility for adjusting the light to improve the surgeon’s visualisation of the operative field. He also insisted – somewhat jokingly given the lack of a real patient – on his right to say when the operation could begin.

However, both teams were ultimately held together and ruled by the surgeon, whose personality exerted a distinct influence over members’ interactions. The hierarchy and gendering of Professor Ellis’s team was immediately apparent. He assumed the role of lead actor in a play, dominating the discourse, ordering other team members around, and looking to his nurse to affirm some of his observations. His requests for instruments were terse and abrupt (‘X please sister!’), and he addressed students as ‘my boy!’ The members of his team colluded with this construction. Juniors addressed him deferentially as ‘Prof.’, and the theatre nurse answered in response to his cues. By contrast, the working of Professor Black’s team appeared more collaborative in nature. Although he remained the focus of discussion and action, he did not perform to his team but rather chatted with them. At times he appeared to negotiate the choice of instrument with his nurse, and issued requests in terms of ‘I’ll need an X.’ His assistants were respectful, but not deferential in manner.

In showing that there was no single template for team working, these differences highlight the impossibility of deriving general insights from individual case studies. Nevertheless, we believe that our observations have a wider applicability. Historians have observed that surgery was a highly personality-driven field, and that through the system of hands-on training of junior staff, surgeons ensured that their methods and ways of working were disseminated to subsequent generations. Ellis and Black were just two of the wider population of surgeons moulded by their respective teachers. In turn, as highly successful, influential surgeons, each had multiple opportunities to mould the next generation. This suggests that the patterns of working that we observed were not unique to them, but were more widely distributed throughout the profession. This impression has been confirmed by the responses of numerous surgical team members (current and retired) with whom we have shared our findings.

For the historian-observer of SBR, the technical nature of surgical expertise was particularly difficult to decode. Both surgeons conducted the operations confidently and without hesitation, handling tissues and manipulating instruments in a manner informed by experience and anatomical knowledge. However it was not until they began to train junior surgeons in the procedure that they articulated the skills and actions involved, thereby making them accessible to the external observer. Some of this teaching was verbal, involving direct instruction in where to cut or dissect. Students were also quizzed, especially about the anatomy of the structures being operated on at the time. At times this was designed to put the trainee on the spot. Surgeons also passed on practical tips, demonstrating subtleties of technique which resisted description in words. Sometimes they used instruments as didactic tools, to point out anatomical features or to trace their course in the air above the operative site. Fingers could also become surgical instruments. A surgeon might say ‘at this point you put your finger in here and do *this’*, demonstrating a manoeuvre without describing it further, and inviting the trainee to continue it

To see Mr John Black’s team operating, view supplementary movie 3 (available at http://dx.doi.org/10.1017/mdh.2013.75).

The nurse participated in the training process by anticipating the instruments needed and holding them ready.

Much teaching related to general aspects of operative technique. Specific aspects of the operation were used to address such fundamental matters as operative posture; how to hold, manipulate and use instruments; how to handle and manipulate tissues; how to tie a suture; and how to assist the primary surgeon, thereby becoming a fully functioning member of the surgical team. At the same time, the surgeons gave advice distilled from their own experience or from that of their own mentors, thereby revealing how expertise passed down the generations. Sometimes they offered personal anecdotes and sometimes more general guidance on how to avoid complications or anticipate and circumvent disaster. In revealing what, precisely, was involved in carrying out an apparently straightforward instruction such as ‘expose the gall bladder’, this teaching not only helped to illuminate the technical content of surgical expertise but also the manner of its acquisition.

## Conclusions: Evaluating SBR

We have argued that the tacit and embodied nature of surgical expertise is impossible to capture from traditional sources such as texts and interviews. In textbook accounts of operations, and even in video recordings made for educational purposes, team-members’ interactions, the roles of anaesthetists and nurses, and the skills and insights required to conduct an operation are effaced from the picture. Yet these aspects are central to the practice of surgery. Arguably it is only by making such matters visible that we can understand how surgery was practised, illuminate its social, technical and educational dimensions, and thereby open up the closed space of the operating theatre to the historian’s gaze.

We believe that SBR is capable of achieving this goal. As a method, it draws on historians’ reproductions of past scientific experiments, applies the material constraints of historical re-enactment, and introduces a historical dimension to SBR as practised within clinical training. Participants’ feedback reveals that initial misgivings about possible lack of realism were short-lived. In post-enactment video review sessions, they repeatedly identified aspects of their behaviour of which they had been wholly unaware at the time, and which they had not mentioned during pre-enactment interviews. Such behaviours included anticipating the needs of other team members; passing instruments unprompted; assisting with surgical techniques; communicating in a variety of verbal and non-verbal ways; and using banter, humour and challenge for educational purposes while operating. Our recordings from multiple perspectives have created a record of these behaviours, and of multiple other aspects of routine surgical and pedagogic practice, which can be readily viewed by those not present during the ‘operation’ itself.

At the same time, our ability to recreate a real operation was limited by the nature of simulation. At one level, every participant was well aware that the ‘operation’ was not real and that there was no actual patient on the operating theatre. The deliberate construction of the event was probably most evident in the absence of bleeding in the hybrid model, coupled with anatomical differences between the pig and the human. At other levels, however, participants described feeling completely immersed in the situation and responding authentically (as they perceived it) to the operation and to one another. Our observation of their behaviour endorses this belief. On one occasion the theatre sister (scrub nurse) in Team 1 angrily shooed an ‘unscrubbed’ team member away when he came too close to her instrument trolley, saying he would contaminate the sterile field. Only later did she remember that the procedure was a simulation and that sterility was not required. This perceived authenticity is in line with simulation research across a wide range of domains, most notably perhaps in the reliance placed upon simulation by both civil and military aviation.

One question raised by our use of SBR to recreate the technical and social aspects of surgical expertise is the extent to which this method can capture the practices of a particular period. Since memories are constructed rather than being retrieved, questions arise about the correspondence between practices enacted now and experienced then. Our focus on team working has allowed us to triangulate our data, inviting team members not only to focus upon their own recollected practices but also reflect upon the perceived authenticity of their colleagues’ behaviour. At the very least, we argue that SBR provides a documentary record of practices which by their nature elude description by other means, and which would otherwise go unrecorded.

So far as we are aware, this is the first time such an approach has been adopted within the history of surgery. Our work to date has focused primarily on the development and refinement of the method. Further research is now required using the documents we have created. This will enable us to build on the above observations about the team-based social and technical nature of surgical expertise. The novelty of this methodological approach brings challenges. For example, how can we make this rich data accessible to other scholars, and how should data analysis be approached? Since social practices in the operating theatre are complex, layered and mediated through multiple modes, written transcripts alone are inadequate. At this stage we do no more than highlight the issue and open it for debate. Our own view is that video recordings could be mapped against a written summary, chronicling the key steps of the operation and providing time codes for specific events and transition points. Further analysis at a micro level could be conducted at a later date, perhaps drawing on the growing body of work around ethnomethodological approaches within the operating theatre.^40^

While this paper has focused on the value of SBR to historians, we believe that it also has potential benefits to contemporary surgery.^41^ As noted above, the landscape of general surgery has undergone profound change over the last two decades, both in the way that surgical teams function, and in the nature of the techniques that they perform. This means that many shared tacit and embodied behaviours are in danger of vanishing, and that valuable skills and expertise may disappear. The almost complete eclipse of open cholecystectomy by laparoscopic surgery has resulted in a generation of consultant surgeons who have rarely performed the open technique. Yet when serious complications arise in laparoscopy, they may have to ‘convert’ to open surgery. In such taxing circumstances, the requirement to perform a technique of which they have little background knowledge or experience is likely to result in considerably poorer outcomes than were achieved a generation earlier, to the ultimate detriment of the patient.

Such concerns are not new. In 1994, in response to the initial surge of laparoscopic surgery (and the resulting, widespread problems of iatrogenic injury), a paper entitled ‘Is there a dilemma in training surgeons in both open and laparoscopic biliary surgery?’ opened a debate in the medical press.^42^ Although the paper itself focused more on the challenge of acquiring new laparoscopic skills rather than the danger of losing the old open ones, a commentary on the paper sounded a cautionary note: ‘in the future, the real problem will lie with the practicing surgeon who is asked to deal with the most difficult urgent biliary tract problems having had little practical experience in more elective situations’.^43^

This problem is even more urgent two decades later. SBR may offer a partial solution, by preserving an endangered set of technical skills which could be drawn upon by surgeons of the future. We have demonstrated that it is still possible to bring together members of longstanding multidisciplinary surgical teams for the purposes of SBR, despite the considerable age of their members. Such opportunities cannot last forever, however, and soon it may no longer be possible to reconstitute full teams from long ago. We believe there is an urgent need to carry out this work while there is still time.

The speed of change in contemporary surgery, and the rapid disappearance of primary source material from the relatively recent past also make it important to capture present-day operative procedures for future historians. Although, at one level, these procedures are becoming widely accessible online, the *social* practices of surgery with which we are concerned are seldom captured. Consequently, there is a strong argument for the periodic recording and archiving of present-day surgery for analysis by future historians.

## Footnotes

1. Kneebone trained as a general and trauma surgeon in the 1980s, learning to perform open cholecystectomy in the manner described below. After completing his specialist training he changed direction and became a general practitioner, then moved to academia to develop the field of surgical education. Woods trained as a veterinary surgeon and worked in general practice before retraining as a historian of medicine.

2. These topics feature heavily in overview accounts of the history of surgery. They have also been subjected to dedicated analysis. Key accounts include: A.J. Harding Rains, *Joseph Lister and Antisepsis* (Hove: Priory Press, 1977); M. Pernick, *A Calculus of Suffering: Pain, Professionalism and Anaesthesia in Nineteenth-Century America* (New York: Columbia University Press, 1985); Lindsay Granshaw, ‘Upon this principle I have based a practice’: The development and reception of antisepsis in Britain, 1867–90’, in J. Pickstone (ed.), *Medical Innovations in Historical Perspective* (London: Macmillan, 1992), 17–46; Roger Cooter, *Surgery and Society in Peace and War: Orthopaedics and the Organization of Modern Medicine, 1880–1948* (Basingstoke: Macmillan, 1993); Emily Mayhew, *The Reconstruction of Warriors: Archibald McIndoe, the Royal Air Force, and the Guinea Pig Club* (London: Greenhill Books, 2004); Stephanie Snow, *Operations without Pain: The Practice and Science of Anaesthesia in Victorian Britain* (Basingstoke: Palgrave MacMillan, 2006); Peter Jones, *A Surgical Revolution: Surgery in Scotland 1837–1901* (Edinburgh: John Donald, 2007).

3. Andrew Warwick, ‘X-rays as evidence in German orthopaedic surgery, 1895–1900’, *ISIS,* 96 (2005), 1–24.10.1086/49154316114797

4. For example: Harold Ellis, *A History of Surgery* (London: Greenwich Medical Media Ltd, 2001); Knut Haeger, *The Illustrated History of Surgery* (London: Harold Starke Publishers, 1988); Nicholas Tilney, *Invasion of the Body: Revolutions in Surgery* (London: Harvard University Press, 2011); J. Kirkup, *The Evolution of Surgical Instruments: An Illustrated History from Ancient Times to the 20th Century* (Novato, California: Norman Publishing, 2006). For a critique of this literature, see Christopher Lawrence, ‘Democratic, divine and heroic: The history and historiography of surgery’ in: C. Lawrence (ed.) *Medical Theory, Surgical Practice: Studies in the History of Surgery* (London: Routledge, 1992), 1–47.

5. For an early sociological analysis of surgical teamwork, see R.N. Wilson, ‘Teamwork in the operating room’, *Human Organisation*, 12 (1954), 9–14. Its contemporary dimensions are discussed by: S. Timmons and J. Tanner, ‘A disputed occupational boundary: Operating theatre nurses and operating department practitioners’, *Sociology of Health and Illness,* 26 (2004), 645–66; R. Finn, ‘The language of teamwork: Reproducing professional divisions in the operating theatre’, *Human Relations,* 61 (2008), 103–30.

6. Charles Webster, *The National Health Service: A Political History*, 2nd edn (Oxford: Oxford University Press, 2002); G. Rivett, *National Health Service History* (http://www.nhshistory.net/, 2008); R. Canter, ‘Impact of reduced working time on surgical training in the United Kingdom and Ireland’, *Surgeon*, 9, Suppl. 1 (2011), S6-7.10.1016/j.surge.2010.11.02021550000

7. S. Wilde and G. Hirst, ‘Learning from mistakes: Early twentieth-century surgical practice’, *Journal of the History of Medicine and Allied Sciences*, 64 (2008), 38–77, uses the diaries of Australian surgeon, Archibald Watson, to describe the culture of ‘learning by ng’ within surgical practice. Other aspects of surgeons’ expertise and their decision making processes are examined in S. Wilde, ‘See one, do one, modify one Prostate surgery in the 1930s’,*Medical History*, 48 (2004), 351–66. Also see Sally Wilde, *The History of Surgery: Trust, Patient Autonomy, Medical Dominance and Australian Surgery, 1890–1940*, available at http://www.thehistoryofsurgery.com/wp-content/uploads/2010/07/book.pdf Thomas Schlich, *Surgery, Science and Industry: A Revolution in Fracture Care, 1950s–90s* (Basingstoke: Palgrave Macmillan, 2002) describes the creation and dissemination of a uniform method of fracture repair by Swiss surgeons in the later 20th century. Julie Anderson, Francis Neary and John Pickstone, *Surgeons, Manufacturers and Patients: A Transatlantic History of Total Hip Replacement* (Basingstoke: Palgrave Macmillan, 2007) situates the development and use of hip replacement techniques within the context of health care costs and priorities, industrial development and patient perspectives.10.1093/jhmas/jrn05518669573

8. Bruno Latour and Steve Woolgar, *Laboratory Life: The Social Construction of Scientific Facts* (London: Sage, 1979); D Baird, *Thing Knowledge: A Philosophy of Scientific Instruments* (London: University of California Press, 2004); Harry Collins and Robert Evans, *Rethinking Expertise* (Bristol: University of Chicago Press, 2007); Harry Collins, *Tacit and Explicit Knowledge* (London: University of Chicago Press, 2010).

9. S. Hirschauer, ‘The manufacture of bodies in surgery’, *Social Studies of Science*, 21 (1991), 279–319; H. Collins, ‘Dissecting surgery: Forms of life depersonalized’, *Social Studies of Science*, 24 (1994), 311–33. See also the responses to Collins by N. Fox, ‘Fabricating Surgery’, M. Lynch, ‘Collins, Hirschauer and Winch: Ethnography, Exoticism, Surgery, Antisepsis and Dehorsification’ and S. Hirschauer, ‘Towards a methodology of investigations into the strangeness of one’s own culture’, *Social Studies of Science*, 24 (1994), 335–89.

10. **I*bid.*; P. Katz, *The Scalpel’s Edge: The Culture of Surgeons* (London: Allyn and Bacon, 1999); T. Moreira, ‘Coordination and embodiment in the operating room’, *Body and Society*, 10 (2004), 109–29; J. Bezemer, A. Cope, G. Kress and R. Kneebone, “‘Can I have a Johann, please?”: Changing social and cultural contexts for professional communication’, *Applied Linguistics Review*, 2 (2011), 313–34; J. Bezemer, G. Murtagh, A. Cope, G. Kress and R. Kneebone, “‘Scissors, please”: The practical accomplishment of surgical work in the operating heater’, *Symbolic Interaction*, 34 (2011), 398–414.

11. J. Lave and W.E. Wenger, *Situated Learning: Legitimate Peripheral Participation* (Cambridge: Cambridge University Press, 1991); T. Moreira, ‘Coordination and embodiment’, *ibid.*; P. Lyon, ‘A model of teaching and learning in the operating theatre’, *Medical Education*, 38 (2004), 1278–87; J. Bezemer, G. Kress, A. Cope and R. Kneebone, ‘Learning in the operating theatre: A social semiotic perspective’, in V. Cook, C. Daly & M. Newman (eds). *Work-Based Learning in Clinical Settings: Insights from Socio-cultural Perspectives* (Abingdon: Radcliffe, 2012), 125–41; R. Prentice, *Bodies in Formation: An Ethnography of Anatomy and Surgical Education* (Durham: Duke University Press, 2013); J. Bezemer, ‘Gesture in operations’, in C. Jewitt (ed.), *Handbook of Multimodal Analysis*, 2nd edn, (London: Routledge, 2013).

12. C. Bosk, *Forgive and Remember: Managing Medical Failure*, 2nd edn (Chicago: University of Chicago Press, 2003).

13. H. Collins, ‘Expert-systems and the science of knowledge’, in W. Bijker, T. Hughes and T. Pinch (eds), *The Social Construction of Technological Systems* (Cambridge, MA: MIT Press, 1987), 329–48; A. Cambrosio and P. Keating, *Exquisite Specificity: The Monoclonal Antibody Revolution* (Oxford: Oxford University Press, 1995), 45–79; Schlich, *op. cit.* (note 7).

14. Kate Fisher, ‘Oral testimony and the history of medicine’ in M. Jackson (ed.) *The Oxford Handbook of the History of Science* (Oxford: Oxford University Press, 2011), 598–616.

15. Hirschauer,‘The manufacture of bodies’ (note 9).

16. For example: Rodney Maingot, *Abdominal Operations* (New York: Appleton-Century-Crofts, 1980); Cholecystectomy (1983, Training video, Royal College of Surgeons of England).

17. G. Lawrence, ‘The ambiguous artefact: Surgical instruments and the surgical past’ in: C. Lawrence (ed.) *Medical Theory, Surgical Practice: Studies in the History of Surgery* (London: Routledge, 1992), 301.

18. For example, C. Timmermann and J. Anderson (eds), *Devices and Designs: Medical Technologies in Surgical Perspective* (Basingstoke: Palgrave Macmillan, 2006).

19. G. Dening, *Mr Bligh’s Bad Language: Passion, Power, and Theatre on the Bounty* (Cambridge: Cambridge University Press, 1992); W.H. Dray, *History as Re-enactment: R.G. Collingwood’s Idea of History* (Oxford: Clarendon Press, 1995); R. Rosenzweig and D. Thelen, *The Presence of the Past: Popular Uses of History in American Life* (New York: Columbia University Press, 1998); J. Thompson, *War Games: Inside the World of Twentieth-Century War Re-enactors* (Washington: Smithsonian Books, 2004); D. Agnew, ‘History’s affective turn: Historical re-enactment and its work in the present’, *Rethinking History*, 11, 3 (2007), 299–312.

20. Alexander Cook, ‘The use and abuse of historical re-enactment: Thoughts on recent trends in public history’, *Criticism*, 46, 3 (2004), 487–96.

21. *Ibid.*, 491.

22. Lawrence Principe, ‘Alchemy restored’, *ISIS*, 102 (2011), 305–12; Lawrence Principe, *Secrets of Alchemy* (London: University of Chicago Press, 2012).

23. HeeringPeter, ‘On Coulomb’s inverse square law’, American Journal of Physics, 60 (1992), 988–994

24. BelloniL., ‘The repetition of experiments and observations: Its value in studying history of medicine (and science)’, Journal of the History of Medicine and Allied Sciences, 25 (1970), 158–167491120410.1093/jhmas/xxv.2.158

25. SibumOtto, ‘Reworking the mechanical value of heat: instruments of precision and gestures of accuracy in early Victorian England’, Studies in History and Philosophy of Science, 26 (1995), 73–106

26. ChangHasok, *Is Water*/article/back/fn-group/fn/p/mixed-citation/inline-formula *? Evidence, Realism and Pluralism* (Dordrecht: Springer, 2012). See also http://www.hps.cam.ac.uk/people/chang/boiling/index.htm

27. P. Heering, ‘Getting shocks: Teaching secondary school physics through history’, *Science & Education*, 9(4) (2000), 363–73; Hasok Chang, ‘How historical experiments can improve scientific knowledge and science education: The cases of boiling water and electrochemistry’, *Science Education* 20 (2011), 317–41; Dietmar Hottecke, Andreas Henke and Falk Riess, ‘Implementing history and philosophy in science teaching: Strategies, methods, results and experiences from the European HIPST Project’, *Science Education*, 21 (2012), 1233–61.

28. R. Kneebone and F. Bello, ‘Surgical simulation’, in R. Riley (ed.), *Manual of Simulation in Healthcare*, (Oxford: Oxford University Press, 2008), 435–48; R. Kneebone and R. Aggarwal, ‘Surgical training using simulation’, *BMJ*, 338 (2009), b1001; V.N. Palter and T.P. Grantcharov, ‘Simulation in surgical education’, *Canadian Medical Association Journal*, 182 (2010), 1191–6.

29. P. Dieckmann, D. Gaba and M. Rall, ‘Deepening the theoretical foundations of patient simulation as social practice’, *Simulation in Healthcare*, 2 (2007), 183–93; D.M. Gaba, ‘The future vision of simulation in health care’, *Quality and Safety in Health Care*, 13, Suppl. 1 (2004), 2–10.10.1097/SIH.0b013e3180f637f519088622

30. Bezemer *et al*, *op. cit.* (note 11) , 125–41; Kneebone and Bello, *op. cit.* (note 28), 435–48; R. Kneebone, ‘Simulation, safety and surgery’, *Quality & Safety in Health Care*, 19, Ergonomics & Safety Supplement (2010), i47-52; E. Kassab, J.K. Tun and R. Kneebone, ‘A novel approach to contextualized surgical simulation training’, *Simulation in Healthcare*, 7 (2012), 155–61.10.1097/SIH.0b013e31824a86db22495386

31. Kneebone, *ibid.*, i47–52; E. Kassab, J.K. Tun, S. Arora, D. King, K. Ahmed, D. Miskovic, A. Cope, B. Vadhwana, F. Bello, N. Sevdalis and R. Kneebone, “‘Blowing up the barriers” in surgical training: Exploring and validating the concept of distributed simulation’, *Annals of Surgery*, 254 (2011), 1059–65.10.1097/SLA.0b013e318228944a21738021

32. J.J. Tang, J. Maroothynaden, F. Bello, R.L. Kneebone. ‘Public engagement through shared immersion: Participating in the processes of research’. *Science Communication*, 2012, 26 November 1075547012466389.10.1177/1075547012466389PMC442865025977603

33. Rodney Maingot, *Abdominal Operations* (New York: Appleton-Century-Crofts, 1980).

34. Cholecystectomy (1983, Training video, Royal College of Surgeons of England).

35. M. Farquharson, B. Moran (eds), *Farquharson’s Textbook of Operative General Surgery* (Hodder Arnold Publication, 2005).

36. Canter, *op. cit.* (note 6), s6–7; J. Temple, ‘Time for training: A review of the impact of the European Working Time Directive on the quality of training’ (http://www.mee.nhs.uk/PDF/14274%20Bookmark%20Web%20Version.pdf, 2010); C. Morris, ‘Reimagining “the firm”: Clinical attachments as time spent in communities of practice’, in V. Cook, C. Daly and M. Newman (eds), *Work-Based Learning in Clinical Settings* (Milton Keynes: Radcliffe Publishing, London, 2012), 11–26.

37. Dixon-WoodsM., YeungK. and BoskC., ‘Why is UK medicine no longer a self-regulating profession? The role of scandals involving “Bad Apple” doctors’, Social Science & Medicine, 73 (2011), 1452–14592197502710.1016/j.socscimed.2011.08.031

38. BracegirdleB., The Wellcome Museum of the History of Medicine: A Part of the Science Museum (London: Science Museum, 1981).

39. From within Imperial College we were able to garner a full set of cholecystectomy instruments together with an anaesthetic machine (Boyle’s machine with Manley ventilator) from the period. Advice and information was provided by curators of and specialist advisers to the Hunterian Museum (Royal College of Surgeons of England), Association of Anaesthetists, Wellcome Collection, Thackray Museum and the Army Medical Museum. Additional material (including scrubs, gowns and caps) were hired from *Film & TV Medical*, which provides an extensive resource of authentic equipment and consumables for drama and documentary production.

40. Bezemer, Cope and Kneebone *op. cit* (note 11), 125–41; Bezemer, Cope, Kress and Kneebone, *op. cit* (note 10), 398–414; J. Hindmarsh and A. Pilnick, ‘The tacit order of teamwork: Collaboration and embodied conduct in anaesthesia’, *The Sociological Quarterly*, 43(2) (2002), 139–64; D. Goodwin, *Acting in Anaesthesia: Ethnographic Encounters with Patients, Practitioners and Medical Technologies* (Cambridge: Cambridge University Press, 2009).

41. KneeboneR. and WoodsA., ‘Bringing surgical history to life’, BMJ, 345 (2012), e81352324966610.1136/bmj.e8135PMC4416206

42. DunhamR. and SackierJ., ‘Is there a dilemma in adequately training surgeons in both open and laparoscopic biliary surgery?’, Surgical Clinics of North America, 74 (1994), 913–9218047949

43. PittR., ‘Commentary: Is there a dilemma in adequately training surgeons in both open and laparoscopic biliary surgery?’, Surgical Clinics of North America, 74 (1994), 9288047949

